# Otorhinolaryngology services during the COVID-19 pandemic in Tanzania

**DOI:** 10.1186/s41182-021-00317-z

**Published:** 2021-03-25

**Authors:** Zephania Saitabau Abraham

**Affiliations:** Department of Surgery, School of Medicine and Dentistry, University of Dodoma, Dodoma, Tanzania

## Abstract

Coronavirus disease-2019 (COVID-19) caused by severe acute respiratory syndrome coronavirus 2 (SARS-CoV-2) remains to be a global pandemic and cause of significant morbidity and mortality. Since the mode of transmission of the virus from one person to another is through respiratory droplets, saliva, and fomites, otorhinolaryngologists are highly exposed to COVID-19 while executing their daily functions. Examination of anatomic sites like the nose and throat exposes the otorhinolaryngologist to possible contact with bodily fluids from the patient. Such examination also requires maintenance of close contact with the patient further increasing the exposure risk. Despite the heightened odds of contracting COVID-19 infection during their routine practice, otorhinolaryngologists in Tanzania have continued managing patients while adhering to the available local guidelines aimed at protecting themselves and also the patients from COVID-19. The aim of this letter it to oversee the current status of otorhinolaryngology services in Tanzania during this era of COVID-19 pandemic.

To the Editor

COVID-19 was declared a global pandemic by the WHO on 11 March 2020, and it has continued to cause many deaths worldwide [1]. In Tanzania, the first case of COVID-19 was confirmed on 16 March 2020 [2]. This disease has continued to affect individuals across all spheres of life, ranging from low to high socioeconomic status. The trend of COVID-19 in Tanzania has been different from other countries. The number of admissions due to COVID-19 has not overwhelmed the health system in Tanzania. This difference could be attributed to climate factors, genetic factors, herd immunity, young population in the country (median age of 18 years) [3], and pre-pandemic cross-reactivity with existing human coronaviruses (HCoVs) [4]; all of which may lead to asymptomatic infections or lower disease incidence. However, the limited capacity of case detection and lack of publicly available data on disease incidence in the country curb these attributions.

The majority of patients with COVID-19 initially visit health care facilities seeking otorhinolaryngologists’ attention due to the clinical presentation (cough, fever, sore throat, runny nose, shortness of breath, nasal obstruction, anosmia, or dysgeusia) manifested by the disease [5–8]. Cognizant of the modes of transmission of the SARS-CoV-2, such as contact with body fluids from the nose and throat [9], and the nature of surgical procedures done regularly, it is clear that otorhinolaryngologists are among the most COVID-19-vulnerable group of health care workers. As such, otorhinolaryngologists should take extra precautions as they continue managing patients and observing WHO recommendations for preventing transmission of the virus.

The risk of COVID-19 exposure to both surgeons and patients increases with diagnostic procedures that bring the surgeon in contact with saliva, mucus, or blood during examination of a patient, whether through traditional methods of using specially designed mirrors, speculums, and laryngoscopy or via endoscopy like nasal endoscopy [6, 9]. The virus’s incubation period is long and unpredictable (0–27 days, mean 6.4 days) [10, 11]; hence, the risk of contracting COVID-19 is higher when healthcare professionals dealing with the oral cavity fail to observe the WHO safety recommendations while unknowingly managing asymptomatic COVID-19 carriers with the potential to transmit the virus.

Hospitals such as Bugando Medical Centre, Benjamin Mkapa Hospital, Mbeya Zonal Referral Hospital, Muhimbili National Hospital, and Kilimanjaro Christian Medical Centre serve the most significant number of patients with otorhinolaryngological conditions in Tanzania. Their otorhinolaryngology departments receive patients with different ear, nose, and throat conditions from various parts of the country. The surgical procedures conducted in these departments vary from minor surgeries such as excision of cystic masses in the nasal cavities to major surgeries such as microlaryngeal surgeries, tonsillectomy, or endoscopic rhinological procedures. The fact that asymptomatic SARS-CoV-2 carriers can transmit the disease [10, 11], the more patients attended by these hospitals in the otorhinolaryngology department, the higher the chances of coming into contact with the asymptomatic COVID-19 carriers within hospital premises.

Currently, there are several available guidelines in managing patients including those with otorhinolaryngological conditions during this pandemic [11, 12]. Majority of the recommendations from the guidelines are easily applicable in our local setting with the exception of a few that may not be possible. Easily executable recommendations include hand hygiene practices, barrier techniques, the use of personal protective equipment such as surgical masks and face shield/goggles, disinfection of surfaces, and sterilization of equipment [1, 12, 13].

Moreover, during the initial phase of COVID-19 transmission, the Ministry of Health in Tanzania offered precautionary guidelines to reduce transmission of the virus, including the postponement of elective surgeries while carrying out only emergency surgeries to protect the community, patients, and staff. Currently, elective surgeries are performed, including those of the throat (tonsillectomy, micro-laryngeal surgeries, laryngectomies) and nose (septoplasty, functional endoscopic sinus surgeries, endoscopic turbinoplasty) have resumed, and this poses a threat towards the transmission of the virus from asymptomatic COVID-19 patients to surgeons and vice versa. This reversal in practice has not resulted in the rise of COVID-19 cases or deaths within the country, which seems puzzling, though may be confounded by the limited publicly available data in our country.

When COVID-19 was reported to be a threat to our country after confirmation of the first case in Tanzania on 16 March 2020, most of the hospitals, including those attending patients with otorhinolaryngological conditions, proposed routine measurement of patient’s/patients’ relatives body temperature using a non-contact infrared thermometer and wearing of masks and hand washing using running water by all people before stepping into hospital premises. These precautions were coupled with a thorough brief history that focused on inquiring about a recent travel to COVID-19-affected place; presence of shortness of breath, sore throat, and cough, and loss of smell/taste. Currently, only a few hospitals are continuing with such practices. The screening protocol was essential in early identification of febrile patients who could be suffering from COVID-19 even though fever alone is not specific to the disease, but when coupled with other symptoms, it increased the index of suspicion for harboring the virus.

The importance of identifying COVID-19-infected patients who at the same time are also suffering from otorhinolaryngological conditions is due to risk of morbidity and mortality post-surgery. Performance of surgical procedures such as total maxillectomy, laryngectomy, or functional endoscopic sinus surgery to a COVID-19-carrier patient may lead to prolonged hospital stay and risk of being intubated that requires an intensive care and thus increasing the chances of being readmitted due to respiratory problems.

It would therefore be ideal to perform routine SARS-CoV-2 tests to all patients admitted in otorhinolaryngology department in the mentioned hospitals as some authors have suggested [12, 13]. An alternative would be to perform a chest radiograph in all cases before being admitted in otorhinolaryngology departments since it can be a preliminary indicator of COVID-19 [1].

It is our duty as otorhinolaryngologists to protect both patients and health care professionals from COVID-19 so as to prevent morbidity and mortality that may emanate from being infected by the virus and also to ensure efficient functioning of the health care system. Clinicians should therefore continue wearing surgical masks, continue regular hand washing and maintain social distance, and reduce non-emergency contact with patients during this era where some patients are presenting with sudden onset of anosmia and/or dysgeusia [6]. All these should be done generally without compromising the care offered to the patient as well as the safety of the surgeon. Other health care workers should also support the surgeons’ effort by wearing protective gadgets such as surgical masks, goggles, and aprons when attending patients. WHO recommends face shields and goggles to be disinfected while the mask and aprons are to be changed and disposed of appropriately after attending every patient.

When COVID-19 was officially announced in Tanzania, congestion of wards was minimized by avoiding unnecessary admissions and avoiding long hospital stay, and even follow-ups post treatment were scheduled after a long span of time, but currently such measures are not emphasized, and this may compound the risk of COVID-19 transmission in Tanzania.

In conclusion, in this era of COVID-19 pandemic, otorhinolaryngologists should continue with routine practice of managing patients with ear, nose, and throat conditions while taking practicable precautions to prevent COVID-19 transmission. Such efforts should go hand in hand with those directed at establishing local guidelines which would ensure safety in delivering health care services to patients in this era of COVID-19 pandemic.

## Data Availability

The information described in this letter may be obtained from the author upon reasonable request.

## Abbreviations

COVID-19: Coronavirus disease-2019
SARS-CoV-2: Severe acute respiratory syndrome coronavirus 2
WHO: World Health Organization
